# The Critical Blood-Sparing Effect of Tranexamic Acid (TXA) in Liposuction: A Systematic Review and Meta-Analysis

**DOI:** 10.1016/j.jpra.2023.01.002

**Published:** 2023-02-02

**Authors:** Myrna Eliann Reinhardt, Sudeep Mutyala, Mykal Gerald, Huaqing Zhao, Vitalina Nova, Sthefano Araya Cambronero, Sameer Patel, Pablo A. Baltodano

**Affiliations:** 1 Albany Medical College, Albany, NY, USA; 2 Fox Chase Cancer Center, Philadelphia, PA, USA; 3 Lewis Katz School of Medicine at Temple University, Philadelphia, PA, USA; 4 Temple University, Charles Library, Philadelphia, PA, USA; 5 Miami Beauty Specialists, Miami, FL, USA

**Keywords:** Liposuction, TXA, Tranexamic Acid, Anti-Fibrinolytic, Liposculpture

## Abstract

**Introduction:**

Tranexamic acid (TXA) has been used to improve bleeding outcomes in many surgical procedures. However, its blood-sparing effect in liposuction is not well established.

**Methods:**

A systematic literature search was performed using PubMed, Embase, Cumulated Index to Nursing and Allied Health Literature (CINAHL), Cochrane Central, ClinicalTrials.gov, and WorldWideScience.org databases from their inception to October 8, 2021, according to Preferred Reporting Items for Systematic Reviews and Meta-Analyses (PRISMA) guidelines. The authors focused on 3 main topics: 1) TXA, 2) liposuction, and 3) complications. We included articles evaluating the potential blood-sparing effects of TXA in liposuction. Studies were excluded if they were systematic review articles or protocol papers, animal studies, conference abstracts, survey studies, or non-English publications.

**Results:**

A total of 711 articles were identified, with 1 retrospective and 4 prospective (3 randomized) studies meeting our inclusion criteria. TXA was used in various forms: administered intravenously either on induction or after the procedure, mixed into the tumescent solution, or infiltrated into the liposuction sites after lipoaspiration. A significantly smaller reduction in hematocrit was noted in the TXA group compared with that in the non-TXA group (p<0.001) despite a significantly greater amount of lipoaspirate removed in the TXA group (p<0.001). Patients in non-TXA cohorts experienced adverse effects (such as seroma and need for transfusion) that were not seen in TXA cohorts.

**Conclusion:**

TXA use in patients undergoing liposuction seems to be associated with a beneficial blood-sparing effect, which may enhance safety in this population. Future studies should aim to determine the optimal route and dosing for TXA in liposuction.

**Evidence Based Medicine:**

Level IV.

## Introduction

Liposuction continues to be one of the most popular cosmetic surgeries performed, with nearly 300,000 procedures performed across both sexes in 2020.^1^ The procedure is typically performed under controlled settings on relatively healthy patients with acceptable risk and safety profiles.^2^ Some of the most common complications include contour irregularity, ecchymosis, hematoma, seroma, surgical site infection, and venous thromboembolism.^3^ In addition to patient safety concerns, these complications are associated with decreased patient satisfaction.^4^ Although techniques have been introduced to minimize these complications (including achieving hemostasis during surgery and using sealants, drains, and compression),^5^ complications surrounding bleeding have remained a significant concern, especially in cases of high-volume liposuction.

Antifibrinolytic pharmacological agents, such as tranexamic acid (TXA) and ε-aminocaproic acid, are known for their utility in minimizing bleeding.^6^ TXA is a synthetic derivative of lysine that reversibly blocks the binding sites of plasminogen, preventing activation of plasmin and enzymatic degradation of the fibrin clot.^7^ The safety and efficacy of TXA has been well characterized in many fields, including cardiac, orthopedic, and gynecologic surgery.^8^ Murphy et al. demonstrated that TXA significantly reduced blood loss and has Level I evidence in the fields of craniofacial and orthognathic surgery.^9^ Although several studies have investigated TXA's beneficial role in various aesthetic surgeries, Level 1 evidence is lacking. Specifically, TXA's blood-sparing effect in liposuction is not well established.

Thus, the aim of this systematic review was to determine the effect of TXA use on blood loss after liposuction procedures and determine whether TXA use in liposuction changes the morbidity profile of the procedure. The authors were also interested in examining the ideal routes of administration and dosing.

## Methods

### Study Identification and Selection

A systematic literature search strategy was developed by our team and a medical librarian (MER, SM, PAB, VN) accordance with Preferred Reporting Items for Systematic Reviews and Meta-Analyses (PRISMA) guidelines. The authors focused on 3 main topics: 1) TXA, 2) liposuction, and 3) complications. The search strategy was developed for PubMed (National Library of Medicine) and was translated to Embase (Elsevier), Cumulated Index to Nursing and Allied Health Literature (CINAHL (EBSCOHost)), and Cochrane Central (Wiley). A gray literature search included ClinicalTrials.gov and WorldWideScience.org. The search included no major limits or date restrictions. The final search was completed on October 8, 2021. The full search details are provided in Appendix A.

PubMed (National Library of Medicine) from inception to October 8, 2021 (62 results)
Embase (Elsevier) from inception to October 8, 2021 (376 results)
CINAHL (EBSCOHost) from inception to October 8, 2021 (41 results)
Cochrane Central (Wiley) from inception to October 8, 2021 (74 results)

The search retrieved 759 studies (206 from gray literature sources). After 48 duplicate studies were found and omitted using Endnote X.20, 711 references were eligible for screening.

Studies were screened by title and abstract by two blinded and independent reviewers (MER, SM). If consensus could not be reached, a third reviewer was consulted. This process was repeated for full-text article screening and article selection. We included articles evaluating potential blood-sparing effects of TXA in liposuction. Systematic review articles or protocol papers, animal studies, conference abstracts, survey studies, and non-English publications were excluded.

### Statistical Analysis

The articles that met our inclusion criteria were reviewed to determine the variables that could be used for a meta-analysis. We identified the following measurements for the review: age, aspirated volume, and hematocrit reduction. Continuous variables were measured as the mean difference and 95% confidence intervals. We did not impute missing data for any outcome. We assessed heterogeneity between studies by estimation of the *I*^2^ statistic and by a formal statistical test to indicate statistically significant heterogeneity. We conducted meta-analysis using a fixed-effect inverse-variance model and performed statistical tests for overall treatment effects between TXA and control. p values of <0.05 were considered statistically significant. All data analyses were performed using Stata 17.0 (StataCorp LLC., College Station, TX).

## Results

**Figure 1:**
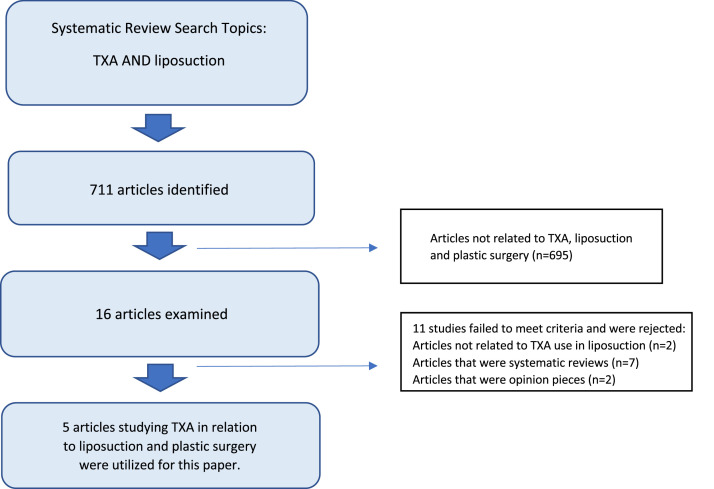
Preferred Reporting Items for Systematic Reviews and Meta-Analyses (PRISMA) flow diagram of articles screened and selected for meta-analysis.

The search identified 711 potentially relevant articles, of which 695 were excluded because they did not relate to TXA and liposuction. We assessed the remaining 16 articles by reviewing their abstracts and manuscripts and applying our inclusion/exclusion criteria. This narrowed our results to 5 relevant publications (Figure 1). The publications included 1 retrospective^10^ and 4 prospective^6^^,^^11^, ^12^, ^13^ (3 randomized^11^, ^12^, ^13^) studies.

TXA was used in various forms: administered intravenously either on induction or after the procedure,^6^ mixed into the tumescent solution,^11^, ^12^, ^13^ or infiltrated into the liposuction sites after lipoaspiration^10^ (Table 1). No local or systemic adverse effects of TXA were observed (including deep venous thrombosis, pulmonary embolism, seizures, and hematoma), and complications were most often found in the non-TXA cohorts. Rodríguez-García et al.^13^ found that the need for blood transfusion was present in 20% of participants in the non-TXA group, whereas no patients in the TXA group required blood transfusions. In the study by Weissler et al.,^10^ 1 patient in the non-TXA group experienced seroma, whereas no seromas were identified in the TXA group. Beneficial effects of TXA found in individual studies included: decreased dermal bleeding,^11^ decreased postoperative ecchymosis/bruising,^10^, ^11^, ^12^ and a decreased total amount of blood in lipoaspirates.^6^^,^^11^

**Table 1:** Included Studies^6^, ^10^, ^11^, ^12^, ^13^: Timing, Route of Administration, and Dosing of TXA.

Study	Route/Timing	Dosing (TXA)
Abboud et al., 2021	IV on induction	0.5 g
	Infiltrated prior to liposuction	0.5 g/L of tumescent solution
Cansancao et al., 2018	IV in preoperative and postoperative periods	10 mg/kg
Fayman et al., 2021	Infiltrated prior to liposuction	0.5 g/500 mL of tumescent solution
Rodríguez-García et al., 2021	Infiltrated prior to liposuction	1 g/L of tumescent solution
Weissler et al., 2021	Infiltrated into liposuction donor sites	3 g of TXA in 75 mL of NaCl 0.9%

**Figure 2:**
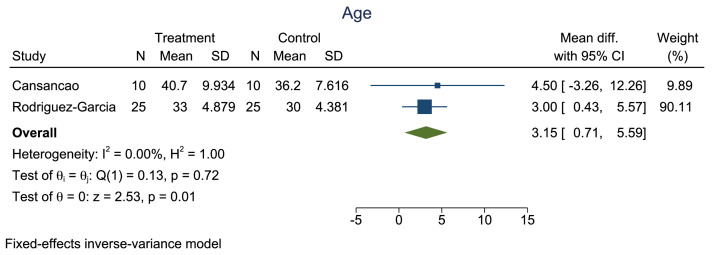
Forest plot of age.

**Figure 3:**
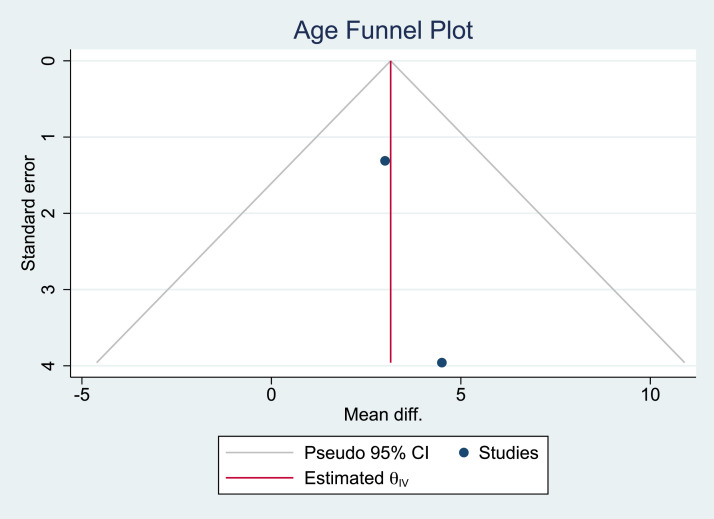
Funnel plot of age.

**Figure 4:**
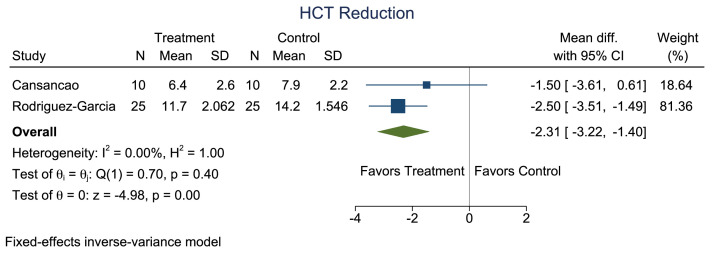
Forest plot of mean difference for the outcome of hematocrit reduction.

**Figure 5:**
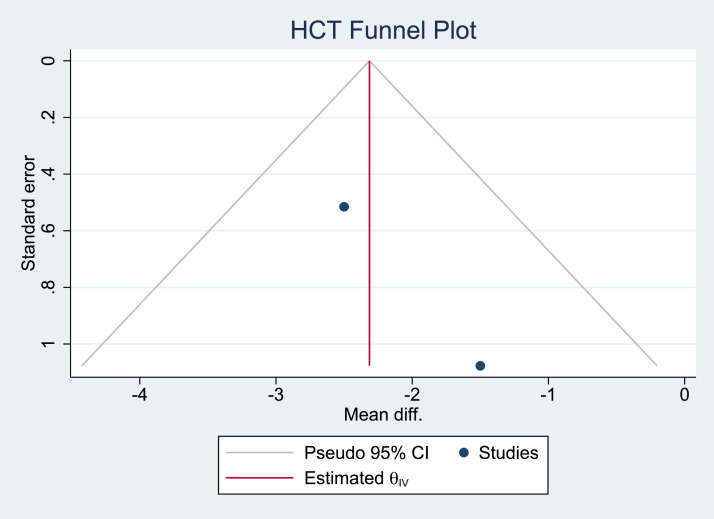
Funnel plot for the outcome of hematocrit reduction.

**Figure 6:**
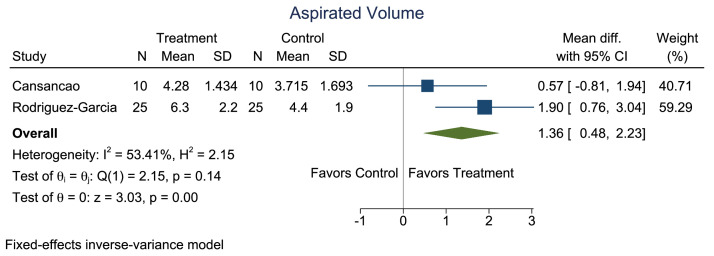
Forest plot of mean difference for the outcome of aspirated volume.

**Figure 7:**
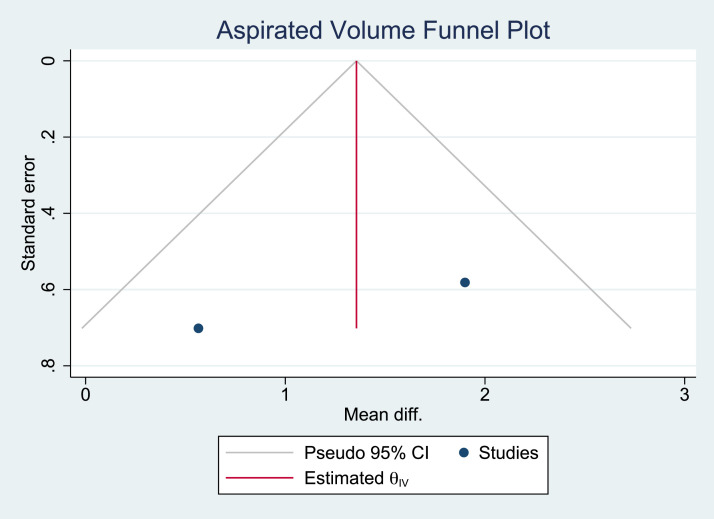
Funnel plot for the outcome of aspirated volume.

Owing to the significant heterogeneity among the 5 studies, only 2 (the studies by Cansancao et al.^6^ and Rodríguez-García et al.^13^) were included in our meta-analysis. A comparison of age showed no significant heterogeneity (*I*^2^ = 0%; Figures 2 and 3). A significantly smaller reduction in hematocrit was noted in the TXA group compared with that in the non-TXA group (p<0.001; Figures 4 and 5) despite a significantly greater amount of lipoaspirate removed in the TXA group (p<0.001; Figures 6 and 7).

## Discussion

Our findings demonstrate that TXA has a beneficial blood-sparing effect when used in liposuction procedures. The meta-analysis suggests that if TXA is used, a significantly greater amount of lipoaspirate may be taken from a given patient, without significant detriment to hematocrit levels. Cansancao et al.^6^ found that the use of TXA could allow for aspiration of 114% more fat, with comparable variation in hematocrit levels (p<0.009). These studies demonstrate that TXA can safely be used in liposuction procedures because the cohorts did not experience any complications classically associated with TXA use.

Blood loss is expected with liposuction procedures, and valid concerns regarding safety have led to advancements in the field (such as infiltration of a saline solution with lidocaine and epinephrine prior to performing liposuction, use of drains, and compression, etc).^14^ Even with our current techniques, blood loss has continued to be a concern and is especially concerning in cases of high-volume liposuction. The practical implications of our findings are significant because decreasing blood loss, especially in cases of high-volume liposuction (which we define as ≥5 L of lipoaspirate), may enhance the safety of this procedure and reduce the incidence of symptomatic postoperative anemia. This is important because postoperative anemia may lead to unplanned emergency room visits and hospital admissions.

TXA's beneficial effect is due to its mechanism of action as an antifibrinolytic. TXA directly prevents degradation of fibrin clots and, thus, is able to slow bleeding.^7^ Our findings correlate with those of previously published studies identifying TXA's positive blood-sparing effect in many different types of procedures. A systematic review of TXA use in plastic surgery found beneficial blood-sparing effects when TXA was used in rhinoplasty, blepharoplasty, breast surgery, body contouring, burn surgery, and microsurgery.^15^ No thrombosis events were associated with TXA use in plastic surgery procedures. Level 1 evidence supports the use of TXA in orthognathic and craniofacial surgery because several trials showing significant reduction in blood loss (p=0.001) in orthognathic surgery and significant reduction in blood loss (p=0.00001) and reduction in blood transfusion (p=0.0001) in craniofacial surgery have been published.^9^ A meta-analysis of TXA in aesthetic plastic surgery procedures found that TXA is associated with a mean of 26.3 mL of blood loss reduction (p<0.001) and suggested a trend toward decreased odds of postoperative hematoma with TXA use (p=0.055).^16^

Very few postoperative complications have been reported with TXA use in esthetic plastic surgery. In facelift surgery, there has been 1 case of preauricular flap necrosis, 1 case of preauricular epidermolysis, and 1 pulmonary embolism reported 4 weeks after surgery.^16^ However, given the short half-life of TXA and its mechanism of action, the authors could not necessarily attribute the pulmonary embolism to TXA administration. Several studies have demonstrated that TXA does not increase the risk of thromboembolic events. The following are a few examples: a study of orthopedic lower limb procedures,^17^ the Aspirin and Tranexamic Acid for Coronary Artery Surgery trial,^18^ and an excellent systematic review, meta-analysis, and meta-regression published by Taeuber et al.^19^ that included 216 trials investigating thromboembolic complications in intravenous (IV) TXA cohorts. Mild side effects of TXA include nausea and diarrhea; however, these effects were observed in 12% of patients in a trial on heavy menstrual bleeding with a dosage of 1 g 4 times daily for 4 days.^20^ With a decrease in dosage, the gastrointestinal symptoms will diminish.^21^

### Route of Administration/Dosing

There are no standard recommendations regarding the route of administration or dosing of TXA in liposuction. Routes of administration, dosing, and timing of administration used in the included studies are summarized in Table 1. The pharmacokinetics and toxicity of TXA have been extensively studied and are well described.^22^

### Systemic Administration

The following recommendations were given by Rohrich et al.^23^ in their review of TXA use in plastic surgery: The most common IV dosages include an initial bolus (usually 10 mg/kg), followed by a constant infusion (1-5 mg/kg/hour) during surgery or two boluses, preoperatively and postoperatively, usually 10 to 15 mg/kg or simply 1 g.^23^ Although these guidelines apply generally to TXA use in plastic surgery, Cansancao et al.^6^ used 10 mg/kg 30 minutes preoperatively and 30 minutes postoperatively, with no detrimental effects noted in the TXA cohort. Thus, we consider this dosing regimen to be appropriate for patients undergoing liposuction. This dose is significantly less than that recommended for patients with massive bleeds (for example in profuse postpartum hemorrhage). Patients in these cohorts may receive up to 10 g over a 7-hour period.^24^

### Local Infiltration

Three studies included in this review used TXA as a component of the tumescent solution.^11^, ^12^, ^13^ These studies used between 0.5 and 1 g of TXA per liter of tumescent solution. The use of TXA in tumescent solution has not been as extensively studied as IV TXA. Additional studies should be performed to further characterize the benefits of this technique in comparison with those of systemic IV TXA. One study (Abboud et al.^11^) combined the use of IV TXA and infiltration of TXA; however, there is not enough evidence to prove that this technique is superior to isolated local infiltration or systemic TXA use. Weissler et al.^10^ were the first to infiltrate TXA into the liposuction donor sites after liposuction was performed. The efficacy of this novel technique will need to be investigated in future studies.

### Limitations and Future Directions

There are few published studies on the use of TXA in liposuction. All of the studies we included have small sample sizes (10-60 patients in a given cohort). Our meta-analysis was also limited by the heterogeneity of the various studies. The 2 studies in our meta-analysis used different routes of TXA administration (Cansancao et al.^6^ administered TXA intravenously and Rodríguez-García et al.^13^ used TXA as part of the tumescent solution). Future randomized and cohort studies should aim to characterize the optimal route for TXA administration in this population.

### Conclusion

TXA use in patients undergoing liposuction seems to be associated with a beneficial blood-sparing effect, which may enhance safety in this population and is especially important in cases of high-volume liposuction. Of the 3 routes of TXA administration identified in this systematic review (administered intravenously either on induction or after the procedure, mixed into the tumescent solution, or infiltrated into the liposuction sites after lipoaspiration), clear guidelines have been published for intravenous TXA use. When using IV TXA, the authors recommend that 10 mg/kg or 1g be given before and after surgery. When mixed into the tumescent solution, the reported studies have used 0.5 to 1 g in 1-L solutions. The use of a combined technique (IV TXA plus TXA mixed in a tumescent solution) is possible but warrants further study. Future studies should aim to characterize the optimal route and dosing for TXA administration in this population.

## Conflict of Interest

The authors declare that they have no conflicts of interest to disclose.

## Figures and Tables

**Table:** 

(“TXA”[tiab] OR “topical TXA”[tiab] OR “IV TXA”[tiab] OR “intravenous TXA”[tiab] OR “intravenous tranexamic acid*”[tiab] OR “tranexamic acid*”[tiab] OR “antifibrinolytic”[tiab] OR “Antifibrinolytic Agents”[MeSH] OR “Antifibrinolytic Agents”[Pharmacological Action] OR “tranexamic acid”[MeSH]) AND (“surgery, plastic”[MeSH] OR “plastic surger*”[tiab] OR “cosmetic surger*”[tiab] OR “cosmetic procedur*”[tiab] OR “liposuction*”[tiab] OR “body sculpt*”[tiab] OR “fat sculpt*”[tiab] OR “fat suction*”[tiab] OR “adipose tissue remov*”[tiab] OR “adipose tissu*”[tiab] OR “body fat*”[tiab] OR “fat”[tiab] OR “fat tissu*”[tiab] OR “fatty tissu*”[tiab] OR “lipectom*”[tiab] OR “lipolysis”[tiab] OR “lipoplast*”[tiab] OR “Adipose Tissue”[MeSH] OR “lipectomy”[MeSH]) AND (“bleed*”[tiab] OR “bruis*”[tiab] OR “surgical blood loss*”[tiab] OR “surgical hemorrhage*”[tiab] OR “VTE”[tiab] OR “venous thromboembol*”[tiab] OR “DVT”[tiab] OR “deep vein thrombos*”[tiab] OR “PE”[tiab] OR “pulmonary embol*”[tiab] OR “deep venous thrombos*”[tiab] OR “pulmonary thromboembol*”[tiab] OR “thrombos*”[tiab] OR “thrombus*”[tiab] OR “vein thrombos*”[tiab] OR “deep vein thrombos*”[tiab] OR “vena cava”[tiab] OR “blood loss, surgical”[MeSH] OR “Ecchymosis”[MeSH] OR “postoperative hemorrhage”[MeSH] OR “contusions”[MeSH] OR “seroma”[MeSH] OR “hematoma”[MeSH] OR “vena cava, superior”[MeSH])

**Table:** 

(txa:ab,ti OR ((topical NEAR/3 txa):ab,ti) OR 'iv txa*':ab,ti OR 'intravenous txa*':ab,ti OR 'tranexamic acid*':ab,ti OR 'tranexamic acid'/exp OR 'antifibrinolytic agent'/exp OR 'antifibrinolytic agent*':ab,ti) AND ('plastic surger*':ab,ti OR 'cosmetic surger*':ab,ti OR 'cosmetic procedur*':ab,ti OR liposuction*:ab,ti OR 'body sculpt*':ab,ti OR 'fat sculpt*':ab,ti OR 'fat suction*':ab,ti OR 'adipose tissu* remov*':ab,ti OR 'body fat*':ab,ti OR 'fat tissu*':ab,ti OR lipectom*:ab,ti OR lipolysis:ab,ti OR lipoplast*:ab,ti OR 'adipose tissu*':ab,ti OR 'plastic surgery'/exp OR 'esthetic surgery'/exp OR 'liposuction'/exp OR 'lipectomy'/exp OR 'adipose tissue'/exp OR 'body fat'/exp OR 'lipolysis'/exp) AND (bleed*:ab,ti OR bruis*:ab,ti OR 'surgical blood loss*':ab,ti OR 'surgical hemorrhag*':ab,ti OR vte:ab,ti OR ((venous* NEAR/3 thromboembol*):ab,ti) OR dvt:ab,ti OR ((pulmonar* NEAR/3 embol*):ab,ti) OR (('deep venous*' NEAR/3 thromb*):ab,ti) OR ((pulmonar* NEAR/3 thromboembol*):ab,ti) OR thromb*:ab,ti OR ((vein* NEAR/3 thromb*):ab,ti) OR (('deep vein*' NEAR/3 thromb*):ab,ti) OR 'vena cava':ab,ti OR 'bleeding'/exp OR 'contusion'/exp OR 'operative blood loss'/exp OR 'venous thromboembolism'/exp OR 'deep vein thrombosis'/exp OR 'lung embolism'/exp OR 'thrombosis'/exp OR 'thrombus'/exp OR 'vein thrombosis'/exp OR 'superior cava vein'/exp OR 'ecchymosis'/exp OR 'postoperative hemorrhage'/exp OR 'seroma'/exp OR 'hematoma'/exp)

**Table:** 

S1 (TXA OR (topical* NEXT TXA) OR (IV NEXT TXA) OR (intravenous* NEXT TXA) OR (intravenous* NEXT tranexamic* NEXT acid*)OR (tranexamic* NEXT acid*) OR (antifibrinolytic*)):ti,ab,kw S2 ((plastic* NEXT surger*) OR (cosmetic* NEXT surger*) OR (cosmetic* NEXT procedur*) OR (liposuction*) OR (bod* NEXT sculpt*) OR (fat* NEXT sculpt*) OR (fat* NEXT suction*) OR (adipos* NEXT tissue* NEXT remov*) OR (adipos* NEXT tissu*) OR (bod* NEXT fat*)OR fat* OR (fat* NEXT tissu*) OR (lipectom*) OR lipolysis* OR (lipoplast*)):ti,ab,kw S3 ((bleed*) OR (bruis*) OR (surgic* NEXT blood NEXT loss*) OR (surgic* NEXT hemorrhag*) OR (contusion*) OR (seroma*) OR VTE OR (venous* NEXT thromboembol*) OR DVT OR (deep* NEXT vein NEXT thrombos*) OR PE OR (pulmonar* NEXT embol*) OR (deep* NEXT venous NEXT thrombos*) OR (pulmonar* NEXT thromboembol*) OR (thrombos*) OR (thrombus*) OR (vein* NEXT thrombos*) OR (deep* NEXT vein* NEXT thrombos*) OR (vena* NEXT cava*) OR (postoperat* NEXT hemorrhag*)):ti,ab,kw S4 S1 AND S2 AND S3

**Table:** 

((MH “Antifibrinolytic Agents+”) OR (MH “tranexamic acid”)) OR (TI(TXA OR “topical TXA” OR “IV TXA” OR “intravenous TXA” OR “intravenous tranexamic acid*” OR “tranexamic acid*” OR “antifibrinolytic*”) OR AB(TXA OR “topical TXA” OR “IV TXA” OR “intravenous TXA” OR “intravenous tranexamic acid*” OR “tranexamic acid*” OR antifibrinolytic*)) AND ((MH “surgery, plastic+”) OR (MH “Adipose Tissue+”) OR (MH lipectomy) OR TI(“plastic surger*” OR “cosmetic surger*” OR “cosmetic procedur*” OR liposuction* OR “body sculpt*” OR “fat sculpt*” OR “fat suction*” OR “adipose tissue remov*” OR “adipose tissu*” OR “body fat*” OR fat* OR “fat tissu*” OR “fatty tissu*” OR lipectom* OR lipolysis OR lipoplast*) OR AB(“plastic surger*” OR “cosmetic surger*” OR “cosmetic procedur*” OR liposuction* OR “body sculpt*” OR “fat sculpt*” OR “fat suction*” OR “adipose tissue remov*” OR “adipose tissu*” OR “body fat*” OR fat* OR “fat tissu*” OR “fatty tissu*” OR lipectom* OR lipolysis OR lipoplast*)) AND ((MH “blood loss, surgical”) OR (MH Ecchymosis) OR (MH “postoperative hemorrhage”) OR (MH “contusions and abrasions”) OR (MH hematoma+) OR (MH “vena cava, superior”) OR (MH “venous thrombosis+”) OR TI(bleed* OR bruis* OR “surgical blood loss*” OR “surgical hemorrhag*” OR contusion* OR seroma* OR VTE OR “venous thromboembol*” OR DVT OR “deep vein thrombos*” OR PE OR “pulmonary embol*” OR “deep venous thrombos*” OR “pulmonary thromboembol*” OR thrombos* OR thrombus* OR “vein thrombos*” OR “deep vein thrombos*” OR “vena cava*”) OR AB(bleed* OR bruis* OR “surgical blood loss*” OR “surgical hemorrhag*” OR contusion* OR seroma* OR VTE OR “venous thromboembol*” OR DVT OR “deep vein thrombos*” OR PE OR “pulmonary embol*” OR “deep venous thrombos*” OR “pulmonary thromboembol*” OR thrombos* OR thrombus* OR “vein thrombos*” OR “deep vein thrombos*” OR “vena cava*”))

**Table:** 

(“tranexamic acid” OR antifibrinolytic*) AND (“plastic surgery” OR “cosmetic surgery” OR “cosmetic procedure” OR liposuction OR liposculpture OR “fat sculpt” OR “fat suction” OR lipectomy OR lipolysis OR lipoplasty) | Completed

**Table:** 

(TXA OR tranexamic* OR antifibrinolytic*) AND (lipectomy OR liposuction) AND (bleed* OR ecchymosis OR hemorrhag*)

## Appendix A

PubMed (National Library of Medicine)

Embase (Elsevier)

Cochrane Central (Wiley)

Cumulated Index to Nursing and Allied Health Literature CINAHL (EBSCOHost)

Clinicaltrials.gov

WorldWideScience.org | limits: remove Energy Technology Data Exchange (ETDEWEB) from source list
